# The association between solid fuel use and oral health: the mediating effect of depressive symptom

**DOI:** 10.3389/froh.2025.1583685

**Published:** 2025-07-21

**Authors:** Hui Jin, Ai-ping Deng, Hua Tian, Mao-Sheng Ran

**Affiliations:** ^1^ Mental Health Center, West China Hospital, Sichuan University, Chengdu, Sichuan, China; ^2^ Institute of Psychiatry, West China Hospital, Sichuan University, Chengdu, Sichuan, China; ^3^ West China School of Nursing, Sichuan University, Chengdu, Sichuan, China; ^4^ Outpatient Department, West China Hospital, Sichuan University, Chengdu, Sichuan, China; ^5^ Lung Cancer Center/Lung Cancer Institute, West China Hospital, Sichuan University/West China School of Nursing, Sichuan University, Chengdu, Sichuan, China

**Keywords:** solid fuel use, periodontal disease, dental cavity, tooth loss, depressive symptoms

## Abstract

**Objectives:**

This study aimed to investigate the associations between solid fuel use and oral health and the mediating effects of depressive symptom.

**Methods:**

64,521 Indian adults were included in this study. Binary logistic regression was performed to evaluate the associations of solid fuel use with tooth loss, dental cavity, and periodontal disease. Mediating analysis was used to investigate the effects of depressive symptom on the associations between solid fuel use and oral health.

**Results:**

Participants who claimed solid fuel use for cooking exhibited an increased risk of periodontal disease (OR: 1.35, 95% CI: 1.29–1.42) after adjusting for potential confounders. There were no significant associations of solid cooking fuel with tooth loss and dental caries. 7.89% of the relationship between solid fuel use and periodontal disease was mediated by depressive symptom.

**Conclusions:**

The use of solid cooking fuel is associated with the increased prevalence of periodontal disease. Depressive symptom mediates the relationship between solid fuel use and periodontal disease. In the management of oral heath, the adverse impact of solid fuel use should be considered. Future studies should further clarify the mechanisms underlying the association between solid fuel and periodontal disease.

## Introduction

Oral health is a major public health concern and essential to human life (1). According to the Global Burden of Diseases Study, oral conditions collectively affect 3.5 billion people worldwide; Of which, untreated caries in permanent teeth is most prevalent (2). Furthermore, Chen et al. reported that 1.1 billion people worldwide experienced severe periodontitis (3).

Solid fuel use, defined as the main reliance on wood, crop residue, coal, or dung for cooking and heating, is a leading cause of household air pollution (HAP) (4). Household solid fuel generates particulate matter (PM), nitrogen dioxide (NO2), and carbon monoxide (CO) (5), which increases the risk of physical diseases such as hypertension, arthritis, anemia, and digestive cancer (5–8). Globally, 2.67 billion people are exposed to HAP from all sources, contributing to 3.9% of total disability-adjusted life years (DALYs) (9).

In the aspects of oral health, exposure to biomass fuels has been demonstrated to show genetic impacts, which is related to antioxidant defense and inflammation (10). Given the abnormal inflammation was involved in the developments of oral diseases, it is possible that there are significant associations between solid fuel use and oral health. For instance, periodontal disease is an inflammatory-related disease, solid fuel use may be a potential risk factor of periodontal disease and therefore may contribute to dental cavity and tooth loss (11, 12).

Depressive symptoms are also an important factor associated with oral health. For instance, Zwick et al. showed that patients with depressive symptoms had poor oral hygiene and oral health status (13). Moreover, Barman et al. reported a dose-response relationship between depressive severity and number of missing teeth (14). Evidence from Korea National Health and Nutrition Examination Survey also showed that participants with depressive symptoms doubled the risk of not making dental visits despite their perceived care needs (15). Meanwhile, solid fuel use has been suggested to be associated with depressive symptoms. Studies on China Health and Retirement Longitudinal Study revealed that solid fuel use was associated with an increased risk of depressive symptoms [odds ratio (OR)] in a sample of 8,803 middle-aged and elderly people (16). A cross-sectional study from India reported that cooking with biomass was linked with increased risk of depressive symptoms and cognitive impairment (17). These findings suggest that depressive symptoms may show a mediating effect on the association between solid fuel use and oral health.

To our knowledge, studies exploring the associations between solid fuel use and oral health are still limited. The Longi­tudinal Aging Study in India (LASI) is nationwide survey, which provides a representative population of Indian adults. We therefore conducted this first study to explore the associations between solid fuel use and oral health (i.e., tooth loss, dental caries, and periodontal disease), and 2) identify the possible mediating role of depression on these associations.

## Methods

### Study population

**Figure 1:**
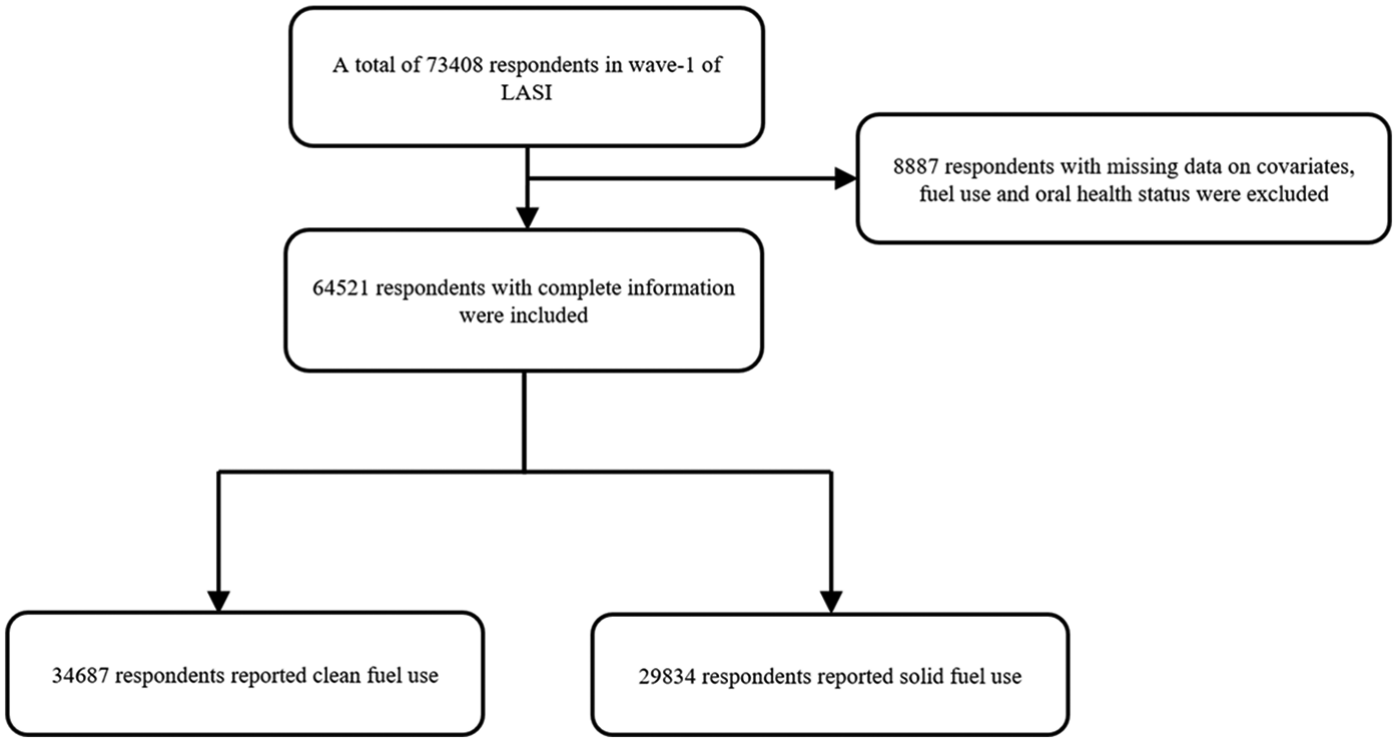
Flowchart of the study. LASI, Longitudinal Ageing Study of India.

The LASI, Wave I, 2017–19 enrolled 73,408 participants aged 45 and older and their spouses regardless of age in the whole country of India. The LASI provides reliable estimates for health measures, economic and social well-being indicators. Details about the survey design, sampling methodology and data collection can be found elsewhere (18). Figure 1 shows the flow chart of participants.

### Solid fuel use

In the harmonized version of the LASI dataset, solid fuel use was defined based on whether the respondent reported their main source of cooking fuel in the household was charcoal/lignite/coal, kerosene, wood/shrub, crop residue, dung cake and etc. Clean fuel use was defined if the housing respondent reported their main source of cooking fuel was electric, biogas, or liquefied petroleum gas.

### Oral health status

In the harmonized version of the LASI dataset, oral health was assessed through self-reported questions. The dataset did not provide specific data on the number of teeth and dental cavities. Instead, tooth loss was dichotomized by whether the respondent had lost all the nature teeth (No/Yes). Dental cavity was identified based on ever being diagnosed with this condition (No/Yes). Periodontal disease was determined by diagnoses of periodontal disease including bleeding gums, swelling gums, and ulcers persisting for more than two weeks (No/Yes) without specifying each symptom separately.

### Depressive symptoms assessment

The 10-item Center for Epidemiologic Studies Depressive Symptom Scale (CESD-10) was utilized to assess depressive symptoms, which exhibits good reliability and validity in older adults (18). Each question is measured based on a Likert scale. “0” indicates the experience rarely or never (less than 1 day); “1” indicates the experience sometimes (1–2 days/week); “2” indicates the experience often (3–4 days/week); and “3” indicates the experience most or all of the time (5–7 days/week). The sum score of the CESD-10 ranges from 0 to 30 with higher scores indicating that the respondent felt more negative experience during the past week.

### Covariates

We incorporated sociodemographic variables including age, sex, marital status (married or partnered/others), economic situation (household consumption per family member). We further adjusted chronic diseases which might be related to oral disease such as diabetes, hypertension, heart disease, lung disease, and thyroid disease. We also included body mass index (BMI), frequency of vigorous physical activity, and self-rated health (SRH) and dental visits. Drinking and smoking status was recorded as “No” and “Yes”. The answer “Yes” meant the respondent had or currently drank or smoked. Dental visits were recorded as “No” and “Yes”. The answer “Yes” meant the respondent had consulted with a dentist in the past year.

### Statistical analysis

Mean and standard deviation was used to describe continuous variables, and proportion was used for categorical variables. A Mann–Whitney U test was used for exploring the between group differences of continuous variables, while a chi-square test was used for categorical variables. We used logistic regression to detect the associations between oral health and solid fuel use with adjusting potential covariates. We used four models in each analysis: model 1 (no adjustment), model 2 (adjusted for age, gender, and BMI), mode 3 (adding indicators of drinking, smoking, SRH, and vigorous physical activity, economic situation, and marital status into model 2), and mode 4 (adding indicators of comorbid diseases: hypertension, diabetes, heart disease, lung disease, and thyroid disease into model 3). We further explored the mediation role of depressive symptom in the association between solid fuel use and oral health. ORs and 95% confidence intervals (CIs) were used to reflect the associations between solid fuel use and oral health. The results with *p* < 0.05 were considered statistically significant. All analyses were performed by R software. Of which, mediation analysis was applied by using mediation package (19). To evaluate the robustness of our findings to unmeasured confounding, E-value analysis was performed (20).

## Results

**Figure 2:**
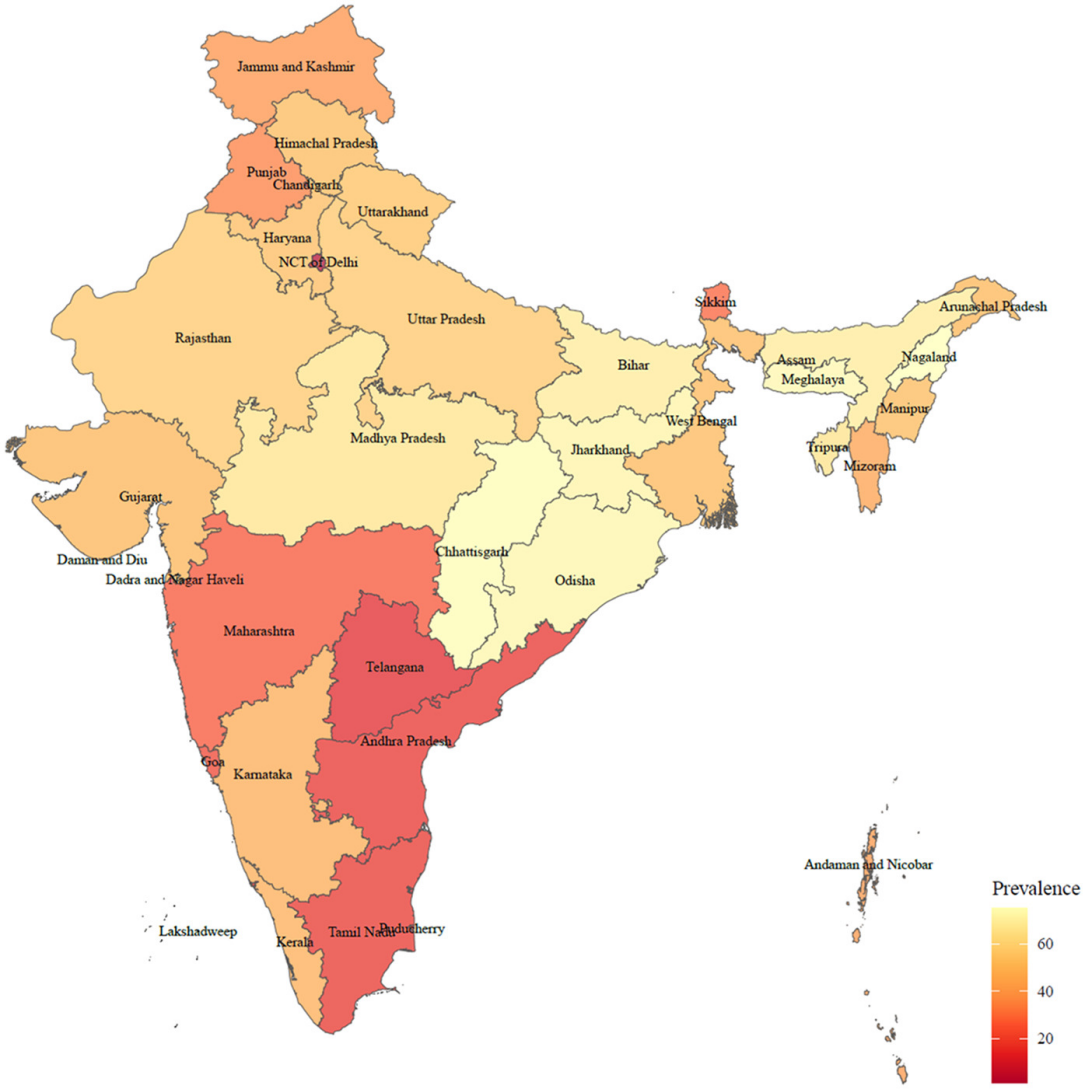
Prevalence of solid fuel use among different Indian states and union territories.

There were 65,421 participants included in our final analysis (Figure 1). The rate of solid fuel use was showed in Figure 2. As given in Table 1, the participants had an average age of 57.7 years old. Compared with participants who did not use solid fuel, participants who used solid fuel were older, and showed higher rates of periodontal disease (17.1%), poor SRH (10.9%), drinking (19.6%), smoking (20.4%), and poorer economic situation (36,200 vs. 57,700).

**Table 1:** Baseline characteristics of participants.

Characteristics	Overall (*n* = 64,521)	Clean fuel use (*n* = 34,687)	Solid fuel use (*n* = 29,834)	*P*
Sex				
Man	27,164 (42.1%)	14,512 (41.8%)	12,652 (42.4%)	0.145
Woman	37,357 (57.9%)	20,175 (58.2%)	17,182 (57.6%)	
Age, years				
Mean (SD)	57.7 (11.5)	57.4 (11.5)	58.0 (11.5)	<0.001
BMI, kg/m^2^				
Mean (SD)	22.9 (4.75)	24.4 (4.81)	21.2 (4.05)	<0.001
Marital status				
Married or partnered	50,217 (77.8%)	27,159 (78.3%)	23,058 (77.3%)	0.002
Others	14,304 (22.2%)	7,528 (21.7%)	6,776 (22.7%)	
Hypertension				
No	46,845 (72.6%)	23,316 (67.2%)	23,529 (78.9%)	<0.001
Yes	17,676 (27.4%)	11,371 (32.8%)	6,305 (21.1%)	
Diabetes				
No	56,861 (88.1%)	28,919 (83.4%)	27,942 (93.7%)	<0.001
Yes	7,660 (11.9%)	5,768 (16.6%)	1,892 (6.3%)	
Lung disease				
No	63,245 (98.0%)	33,955 (97.9%)	29,290 (98.2%)	0.010
Yes	1,276 (2.0%)	732 (2.1%)	544 (1.8%)	
Heart disease				
No	62,342 (96.6%)	33,183 (95.7%)	29,159 (97.7%)	<0.001
Yes	2,179 (3.4%)	1,504 (4.3%)	675 (2.3%)	
Thyroid disease				
No	62,466 (96.8%)	33,106 (95.4%)	29,360 (98.4%)	<0.001
Yes	2,055 (3.2%)	1,581 (4.6%)	474 (1.6%)	
Depression				
No	37,088 (57.5%)	20,848 (60.1%)	16,240 (54.4%)	<0.001
Yes	27,433 (42.5%)	13,839 (39.9%)	13,594 (45.6%)	
Economic situation, rupees				
Mean (SD)	47,700 (58,000)	57,700 (61,600)	36,200 (51,000)	<0.001
Periodontal disease				
No	55,229 (85.6%)	30,489 (87.9%)	24,740 (82.9%)	<0.001
Yes	9,292 (14.4%)	4,198 (12.1%)	5,094 (17.1%)	
Dental cavity				
No	51,918 (80.5%)	27,765 (80.0%)	24,153 (81.0%)	0.004
Yes	12,603 (19.5%)	6,922 (20.0%)	5,681 (19.0%)	
Tooth loss				
No	60,564 (93.9%)	32,616 (94.0%)	27,948 (93.7%)	0.066
Yes	3,957 (6.1%)	2,071 (6.0%)	1,886 (6.3%)	
Vigorous physical activity				
Everyday	15,602 (24.2%)	7,202 (20.8%)	8,400 (28.2%)	<0.001
More than once a week	4,603 (7.1%)	1,783 (5.1%)	2,820 (9.5%)	
Once a week	2,415 (3.7%)	1,178 (3.4%)	1,237 (4.1%)	
One to three times a month	3,315 (5.1%)	1,542 (4.4%)	1,773 (5.9%)	
Hardly ever or never	38,586 (59.8%)	22,982 (66.3%)	15,604 (52.3%)	
SRH				
Excellent	2,678 (4.2%)	1,721 (5.0%)	957 (3.2%)	<0.001
Very good	12,796 (19.8%)	7,491 (21.6%)	5,305 (17.8%)	
Good	25,615 (39.7%)	13,975 (40.3%)	11,640 (39.0%)	
Fair	17,459 (27.1%)	8,778 (25.3%)	8,681 (29.1%)	
Poor	5,973 (9.3%)	2,722 (7.8%)	3,251 (10.9%)	
Drinking status				
No	53,855 (83.5%)	29,854 (86.1%)	24,001 (80.4%)	<0.001
Yes	10,666 (16.5%)	4,833 (13.9%)	5,833 (19.6%)	
Smoking status				
No	53,737 (83.3%)	29,983 (86.4%)	23,754 (79.6%)	<0.001
Yes	10,784 (16.7%)	4,704 (13.6%)	6,080 (20.4%)	

BMI, body mass index; SD, standard deviation; SRH, self-rated health.

In the unadjusted model, significant associations were found between solid fuel use and dental cavity (OR: 0.94, 95% CI: 0.91–0.98, *P* < 0.01), and between solid fuel use and periodontal disease (OR: 1.50, 95% CI: 1.43–1.56, *P* < 0.01). No significant association was observed between solid fuel use and tooth loss. After fully adjustment of potential confounding factors, participants who reported solid fuel use had significantly increased odds of periodontal disease (OR: 1.35, 95% CI: 1.29–1.42, *P* < 0.01). However, there were no significant associations between solid fuel use and tooth loss and dental cavity (Table 2).

**Table 2:** The relationship between solid fuel use and oral health.

Oral health outcomes	Model 1	Model 2	Model 3	Model 4
Clean fuel use	Reference	Reference	Reference	Reference
Solid fuel use				
Periodontal disease	1.50 (1.43–1.56)	1.41 (1.35–1.48)	1.32 (1.27–1.39)	1.35 (1.29–1.42)
Dental cavity	0.94 (0.91–0.98)	0.98 (0.94–1.02)	0.95 (0.91–0.99)	0.97 (0.93–1.01)
Tooth loss	1.06 (1.00–1.13)	0.95 (0.89–1.02)	0.97 (0.90–1.04)	0.97 (0.90–1.04)

Model 1: no adjustment.

Model 2: adjusted for age, gender, and BMI.

Model 3: adjusted for model 2 plus drinking, smoking, dental visits, SRH, and vigorous physical activity, economic situation, marital status.

Model 4: adjusted for model 3 plus hypertension, diabetes, heart disease, lung disease, thyroid disease.

**Figure 3:**
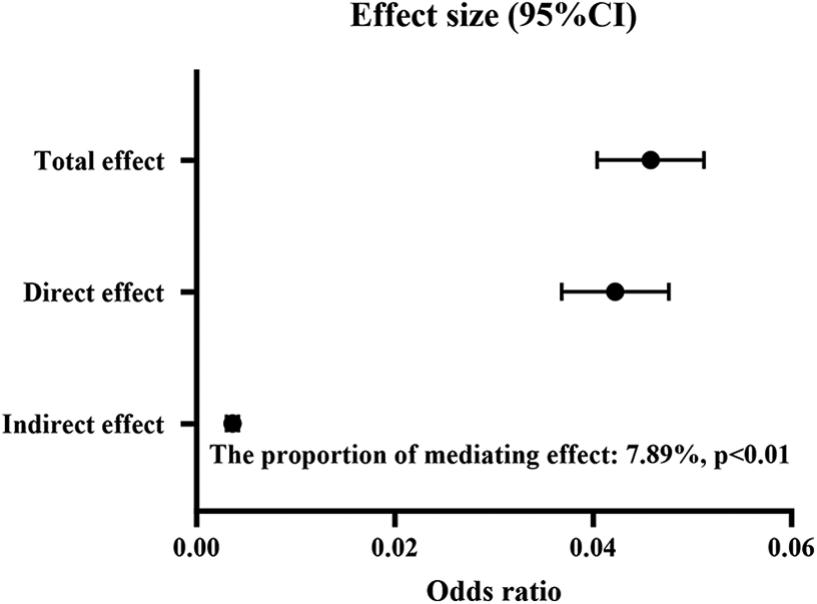
The mediating role of depression between solid fuel use and periodontal disease. CI, confidence interval.

For the mediation analysis, the total effect of solid fuel use on periodontal disease was 0.046 (95% CI: 0.040–0.051, *P* < 0.01), and the direct effect of solid fuel use was 0.042 (95% CI: 0.037–0.048, *P* < 0.01). Depressive symptoms (effect: 0.004, 95% CI: 0.003–0.004, *P* < 0.01) exhibited statistically significant mediating effect on the association between solid fuel use and periodontal disease (Figure 3). 7.89% of the association between solid fuel use and periodontal disease was mediated by depressive symptoms. The *E*-value was 2.59 for the indirect path and 1.67 for the direct path, indicating that the results are moderately robust to unmeasured confounding.

## Discussion

Our study explored the associations between solid fuel use and oral health in a large-scale and representative sample in India, and revealed a significant association between solid fuel use and periodontal disease. Furthermore, depressive symptoms exhibited a significant mediation effect on this association. Of which, 8.45% of the total effect was mediated by depressive symptoms.

The results of this study showed that there was 46.2% of the participants who still used solid fuel for cooking in India. People who used solid fuel showed higher percentage of periodontal disease (17.1%) compared with those who used clean fuel (12.1%), suggesting a possible link between solid fuel related pollution and oral health. Previous studies have also reported the possible associations of PM, NO_2_, and CO which can be generated from household solid fuel with oral health. For instance, Zierold et al. reported that exposure to coal-burning power increased the odds of gingiva symptoms (OR: 2.46, 95% CI: 1.46–4.15) (21). Furthermore, a study conducted among 56,456 outpatient visits of periodontitis show that NO_2_ exposure is a risk factor of periodontitis outpatient visits (22). In addition, Marruganti et al. reported that per 5-μg/m^3^ increase of PM10 increased the risk of periodontitis (OR: 1.17, 95% CI: 1.11–1.24) in Korean population (*n* = 42,020) (23).

To our knowledge, this study, for the first time, revealed a significant association between solid fuel use and periodontal disease. The mechanisms underlying this association are complicated. The elevated inflammatory responses and increased oxidative stress (OS) resulting from PM, NO_2_, and CO which can be generated from household solid fuel may be involved. Animal experiments prove that the generation of PM2.5 can aggravate OS and inflammatory response through the Nrf2/NF-kB signaling pathway (24). Clinically, there are also similar findings in clinical trials. One study in patients with kidney disease indicates that the periodontal disease is associated with higher level of high-sensitivity CRP (25). Moreover, periodontal disease was associated with elevated white blood cell count and inflammatory cytokines like interleukin-1, 6 and 8, and tumour necrosis factor (26). It is reasonable to speculate that exposure to the products of solid fuel use such as PM2.5 increases inflammatory response, contributing to the destruction of periodontal tissues (27). The effects of changes in immune function should also be considered. Previous study suggested that the emission of solid fuel might result in the modulation of the innate immune system and increased susceptibility to infection (28). When exposed to biomass generations, the deleterious health effect might lead to the aggravation of the main pathogenesis of periodontal disease: the infection of bacteria (29).

This study showed that there was significant mediating effect of depressive symptoms on the association between solid fuel use and periodontal disease. Regarding the association between solid fuel and depression, the results of this study showed a higher rate of depressive symptoms in individuals who used solid fuel compared with those who used clean fuel (45.6% vs. 39.9%). A meta-analysis shows that people exposed to solid fuel related HAP has a 22% higher risk of depressive symptoms (30). These findings suggest that solid fuel use may be a significant factor in the developments of depressive symptoms. Abnormal OS may play an important role in this association. The OS contributes to increased production of reactive oxygen species (ROS). The imbalance between antioxidative defenses and ROS causes disruptions in brain functions and neuronal signaling abnormalities, which involves in the development of depression (31). Regarding the associations between depression and oral health, previous studies reported that individuals with depressive symptom were more likely to show poor oral hygiene behavior compared with those without depressive symptoms (32). Nascimento et al. showed that people with depressive symptoms increased the risk of periodontitis (risk ratio: 1.19, 95% CI: 1.04–1.36) (33). In addition, people with severe depressive symptoms had increased odds of mild periodontitis compared to non-symptomatic counterparts (OR = 2.20, 99% CI: 1.03–4.66) (34). These may partly explain the mediating effects of depressive symptoms on the association between solid fuel use and periodontal disease.

This study fulfills the research gap in the association between oral health and solid fuel use, and sheds light on the prevention of oral disease. These findings suggest early interventions toward mental health should therefore benefit oral health in solid fuel users. Moreover, the transition from the use of fossil fuels into clean fuels have significant benefit for public health, especially in northern India, where our study indicates a higher prevalence of solid fuel use. Research conducted in India showed that the adoption of clean cooking fuels in India remained limited, with only about 40% of the wealthiest households relying exclusively on them. The widespread use of solid fuels continues to pose significant environmental and health risks (35). Therefore, reducing reliance on solid fuels for cooking like improving kitchen ventilation and promoting the use of improved biomass stoves should lead to considerable improvements in both oral hygiene and mental health outcomes.

There are limitations in our study. First, the data about depressive symptoms are self-reported. Therefore, recall bias is inevitable and may lead to underreporting or misclassification, which could attenuate the observed associations. Further research will incorporate clinical examinations or dental records to enhance the robustness of the study. Second, for the evaluation of oral health, there are no information about the number of teeth and dental cavities, detailed symptoms of periodontal disease, which hinders further exploration. Furthermore, the lack of detailed data about cooking duration, exactly type of fuel may provide biased estimates of the results. Third, the participants of our study were middle-aged and older Indians, which may limit the generalizability of our findings to other age groups or countries with different healthcare systems and environmental exposures. Fourth, the short time frame of the CESD-10 limits its ability to capture the long-term psychological impact of solid fuel exposure. Fifth, although sensitivity analyses suggest moderate robustness to unmeasured confounding, the overall rigor of the findings could be further improved by incorporating more comprehensive covariate adjustment. Future studies should incorporate longitudinal study designs and clinical records, structured clinical interviews and longitudinal assessments for the evaluation of depressive symptoms. Moreover, expanding the demographic scope beyond middle-aged and older Indians will enhance the generalizability of the findings.

## Conclusion

This study shows that the use of solid cooking fuel is associated with the increased prevalence of periodontal disease. Depressive symptoms mediate the relationship between solid fuel use and periodontal disease. Future studies are needed to further clarify the mechanisms between solid fuel use and periodontal disease. The culture-specific health policies and interventions should be developed to reduce the solid fuel use and improve peoples' mental health and oral health.

## Data Availability

The original contributions presented in the study are included in the article/Supplementary Material, further inquiries can be directed to the corresponding author.
